# Three-dimensional changes of a porcine collagen matrix and free gingival grafts for soft tissue augmentation to increase the width of keratinized tissue around dental implants: a randomized controlled clinical study

**DOI:** 10.1186/s40729-023-00482-2

**Published:** 2023-06-16

**Authors:** Ausra Ramanauskaite, Karina Obreja, Katharina Melissa Müller, Carla Schliephake, Johanna Wieland, Amira Begic, Iulia Dahmer, Puria Parvini, Frank Schwarz

**Affiliations:** Department of Oral Surgery and Implantology, Johann Wolfgang Goethe-University, Carolinum, Frankfurt an Main, Germany

**Keywords:** Dental implants, Free gingival graft, Keratinized mucosa, Soft tissue grafting, Three-dimensional analysis

## Abstract

**Background:**

Emerging clinical data points to the relevance of the presence of keratinized tissue (KT). Although apically positioned flap/vestibuloplasty along with free gingival graft (FGG) is considered as a standard intervention for augmenting KT, substitute materials appear to be a viable treatment alternative. So far, there is a lack of data investigating the dimensional changes at implant sites treated with soft-tissue substitutes or FGG.

**Aim:**

The present study aimed at comparing three-dimensional changes of a porcine derived collagen matrix (CM) and FGG for increasing KT at dental implants over a 6-month follow-up period.

**Materials and Methods:**

The study enrolled 32 patients exhibiting deficient KT width (i.e., < 2 mm) at the vestibular aspect who underwent soft tissue augmentation using either CM (15 patients/23 implants) or FGG (17 patients/31 implants). The primary outcome was defined as tissue thickness change (mm) at treated implant sites between 1- (S0), 3- (S1), and 6-months (S2). Secondary outcomes considered changes of KT width over a 6-month follow-up period, surgical treatment time, and patient-reported outcomes.

**Results:**

Dimensional analyses from S0 to S1 and from S0 to S2 revealed a mean decrease in tissue thickness of − 0.14 ± 0.27 mm and − 0.04 ± 0.40 mm in the CM group, and − 0.08 ± 0.29 mm and − 0.13 ± 0.23 mm in the FGG group, with no significant differences noted between the groups (3 months: p = 0.542, 6 months: p = 0.659). Likewise, a comparable tissue thickness decrease was observed from S1 to S2 in both groups (CM: − 0.03 ± 0.22 mm, FGG: − 0.06 ± 0.14 mm; p = 0.467). The FGG group exhibited a significantly greater KT gain after 1, 3 and 6 months compared to the CM group (1 month: CM: 3.66 ± 1.67 mm, FGG: 5.90 ± 1.58 mm; p = 0.002; 3 months: CM: 2.22 ± 1.44; FGG: 4.91 ± 1.55; p = 0.0457; 6 months: CM: 1.45 ± 1.13 mm, FGG: 4.52 ± 1.40 mm; p < 0.1). Surgery time (CM: 23.33 ± 7.04 min.; FGG: 39.25 ± 10.64 min.; p = 0.001) and postoperative intake of analgesics were significantly lower in the CM group (CM: 1.2 ± 1.08 tablets; FGG: 5.64 ± 6.39 tablets; p = 0.001).

**Conclusions:**

CM and FGG were associated with comparable three-dimensional thickness changes between 1 and 6 months. While a wider KT band could be established with FGG, the use of CM significantly reduced surgical time and patients´ intake of analgesics.

**Graphical abstract:**

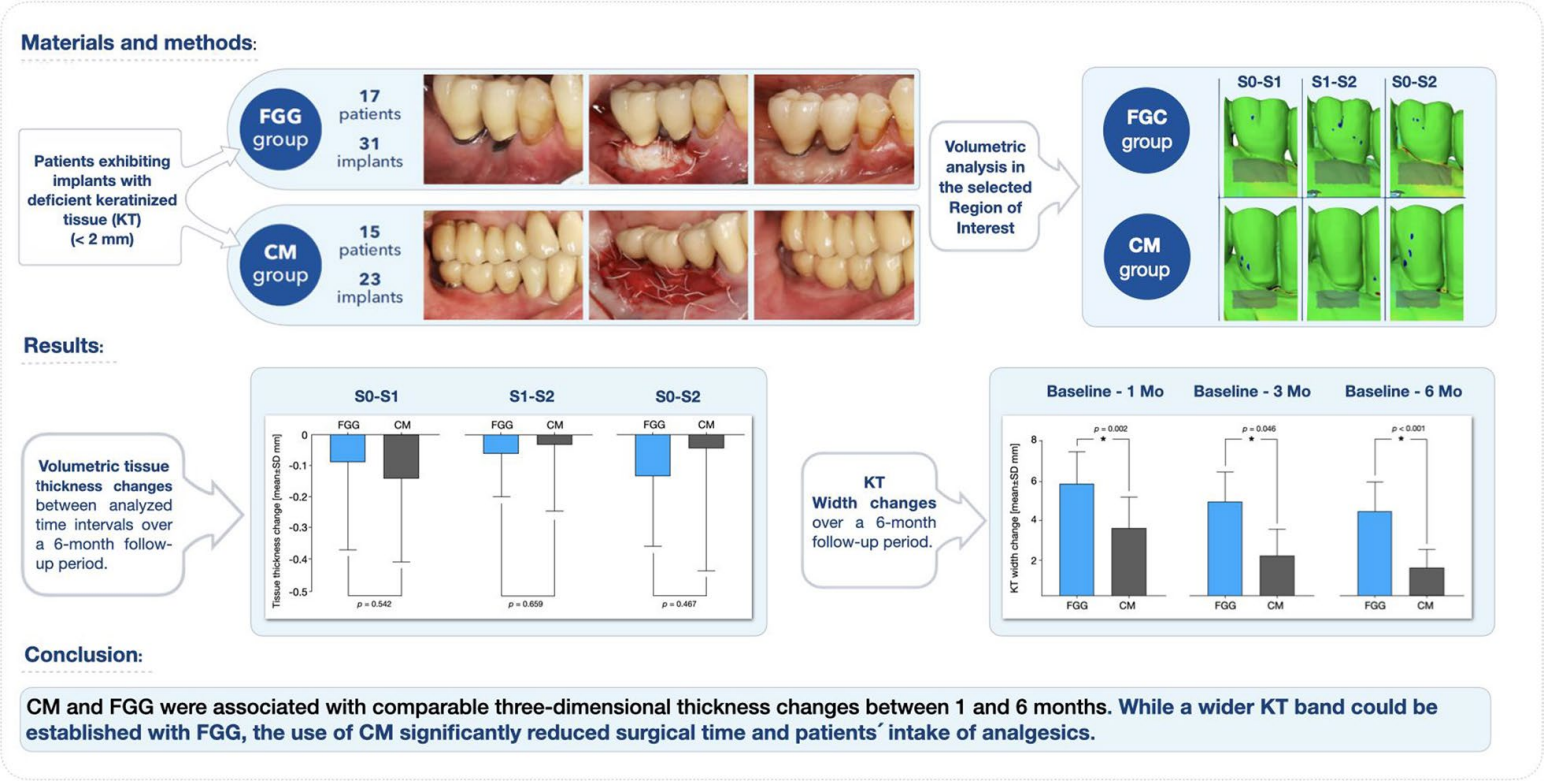

## Introduction

Emerging scientific evidence points to the relevant clinical role of keratinized tissue (KT) in the maintenance of health and stability of peri-implant tissues [1, 2]. A reduced width of KT (i.e., < 2 mm) at implant sites has been associated with increased plaque accumulation, soft-tissue inflammation, greater patient discomfort, mucosal recession, marginal bone loss and an increased prevalence of peri-implantitis [2]. In light of these findings, when a reduced amount of KT coincides with pathological changes in the peri-implant mucosa, surgical interventions aimed at increasing the width of peri-implant KT should be considered [1]. In fact, as shown in one previous meta-analysis, the establishment of KT at implant sites exhibiting a lack of or reduced KT dimensions resulted in significantly greater improvements in mucosal- and plaque indices as well as more stable marginal bone levels compared to non-augmented sites [3].

Currently, the apically positioned flap along with the free gingival grafts (FGG) constitutes the most predictable technique for establishing peri-implant KT [4]. Despite the technique’s high success and predictability, the use of FGG is inevitably accompanied by an increased patient discomfort, a pronounced postoperative shrinkage of the graft and compromised esthetic outcomes [5, 6]. Considering the aforementioned limitations, soft-tissue substitutes have been introduced as alternatives to autogenous tissues [7, 8]. Indeed, based on the estimations of one recent meta-analysis, the use of soft-tissue substitutes of xenogeneic origin appear to be a promising alternative to autogenous grafts, due to a similar increase in peri-implant KT width, but a reduced patient morbidity, shorter surgical time and superior esthetic outcomes [9].

Along these lines, it is important to note emerging evidence associating mucosal thickness with peri-implant tissue health and stability. In fact, implant sites featuring a thin peri-implant mucosa (defined as tissue thickness < 2 mm or categorized based on transparency of the periodontal probe) have been shown to undergo a more pronounced marginal bone loss during the initial remodeling phase, be more susceptible to peri-implant diseases and present inferior esthetic outcomes [10–12]. Accounting for the considerably high three-dimensional (3D) thickness changes of FGG occurring within the first 3-months (71.8%) [6], it might be assumed that soft-tissue substitutes undergo less pronounced 3D alterations, subsequently leading to more predictable outcomes.

So far, clinical data comparing the dimensional changes at implant sites treated with soft-tissue substitutes or FGG to increase the peri-implant keratinized mucosa is lacking. Therefore, the aim of this randomized controlled clinical study was to assess and compare 3D changes of a porcine derived collagen matrix and FGG for increasing KT at dental implants over a 6-month follow-up period.

## Materials and methods

For this randomized controlled two-arm clinical study, a total of 34 fully or partially edentulous patients exhibiting deficient KT width (i.e., < 2 mm) at the vestibular aspect of 56 implants were included. All patients had attended the Department of Oral Surgery and Implantology, Goethe University, Frankfurt, Germany, and were subjected to KT grafting procedures at either second-stage surgery or implants in function as noted during routine yearly maintenance appointments. All surgical procedures were carried out between December 2020 and February 2022.

Each patient was given a detailed information of the study protocol and was required to sign an informed consent form that authorized the collection of personal data and performance of digital and clinical evaluation. The study protocol was in accordance with the Helsinki Declaration of 1975 (revised in August 2018), and approved by the Ethics Committee of the Goethe University (No. 20-541). The reporting of the present study considered the STROBE statement.

### Inclusion criteria

The inclusion criteria considered patients who signed and approved the consent form. Patients had to meet the following criteria:

1. minimum age of 18 years,
2. edentulous or partially edentulous patients who had undergone dental implant surgery at grafted and/or non-grafted (i.e., pristine) sites,
3. patients exhibiting KT width of < 2 mm at the vestibular aspect of the implant subjected to surgical intervention either at the time of second-stage surgery (i.e., 3 to 5 months following submerged healing) or implants in function,
4. presence of peri-implant tissue health, and
5. adequate oral hygiene as evidenced by plaque index (PI) < 1.

### Exclusion criteria

The exclusion criteria considered patients who presented with:

1. general contraindications for dental and surgical treatments,
2. uncontrolled diabetes (HbA1c > 7),
3. autoimmune and/or inflammatory diseases of the oral cavity,
4. active periodontal disease,
5. pregnant or lactating women,
6. smokers (≥ 10 cigarettes per day), and
7. malpositioned implants.

### Sample size calculation

Due to a lack of similar data in the literature, an a priori sample size calculation based on the primary outcome measure (i.e., 3D changes in tissue thickness) was not feasible. However, with the given sample size (15 and 17 patients in the CM and FGG groups, respectively) an effect size of d = 1.2 can be recognized with a power of 80% at a significance level of alpha 1.67% (obtained through Bonferroni correction due to several three t-tests being performed, one for each time point) (PASS 2022, Version 22.0.3). Assuming a standard deviation of 0.3 for the tissue thickness change allows to recognize minimal mean difference of 0.36 between the groups.

### Treatment procedures

Four calibrated experienced clinicians (PP, KO, AB, AR) performed all surgical procedures. After the administration of local anesthesia (2% articaine, 1:100.000 epinephrine), implant sites undergoing a simultaneous second-stage surgery were uncovered and healing abutments of appropriate dimensions were inserted. Afterward, the recipient bed was prepared using a 15-stainless steel blade by performing a horizontal split-thickness incision at the mucogingival junction (MGJ) on the buccal aspect of the implants. In the absence of KT at the recipient area, the entire mucosa at the implant’s buccal aspect was raised. The mucosa was apically positioned and fixed to the periosteum with 4/0 non-resorbable monofilament sutures (Cytoplast PTFE, Osteogenics Biomedical, Lubbock, USA).

At this point, the treatment groups were assigned by means of envelopes containing a code derived from a randomisation list. Based on the assignment, patients were treated with either a porcine collagen matrix (Geistlich Mucograft, Geistlich Biomaterials, Wolhusen, Switzerland; CM) or FGG.

**Fig. 1:**
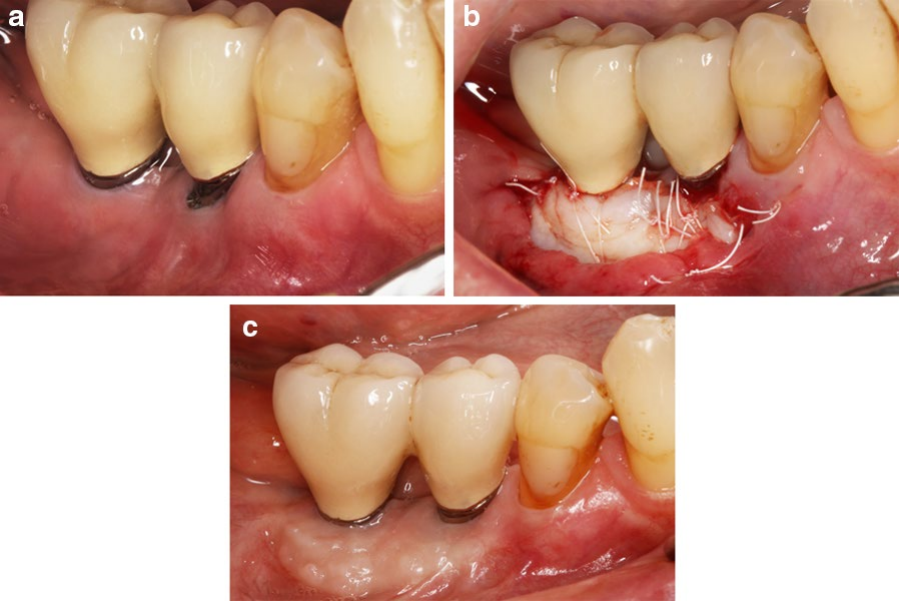
Surgical procedure in the FGG group: **a** preoperative intraoral view showing a lack of keratinized tissue at implants 45 and 46. **b** postoperative view depicting the fixation of FGG at the recipient site. **c** intraoral view of the surgical site after 6 months

In the CM group, the matrix was trimmed to the required dimensions and fixed to the periosteum with interrupted and mattress sutures with 4/0 non-resorbable PTFE monofilament (Cytoplast PTFE). The spongious layer of CM was positioned towards the recipient bed and the compact layer faced the outwards (Fig. 1).

**Fig. 2:**
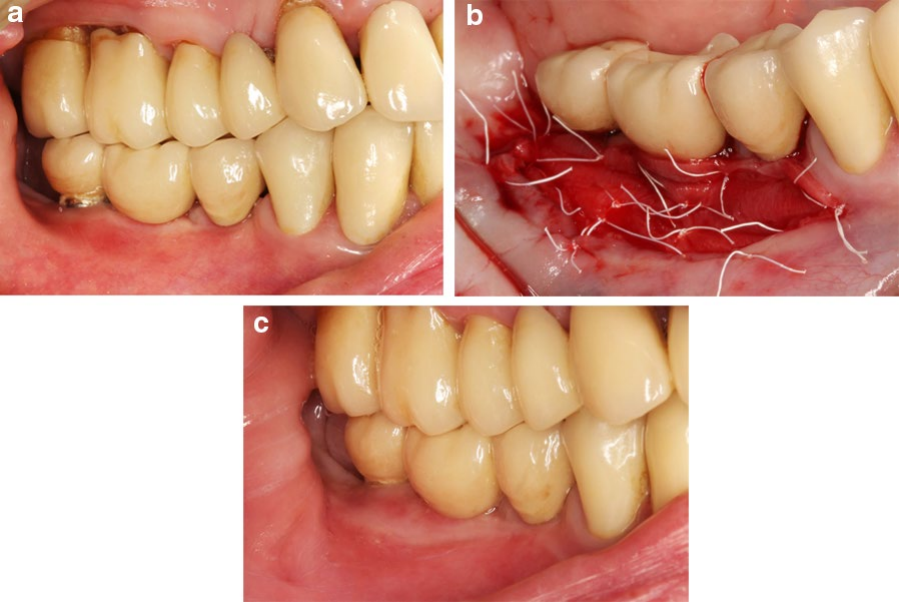
Surgical procedure in the CM group: **a** preoperative intraoral view indicating a lack of keratinized tissue at implants 35 and 37. **b** Postoperative view showing the fixation of CM at the recipient site. **c** Intraoral view of the surgical site after 6 months

In the FGG group, the dimensions of the pre-determined graft size were outlined in the palatal mucosa with a 15-stainless steel blade. A 1.0- to 1.5-mm-thickness FGG was harvested from the donor area located between the first premolar and the first molar and 2 mm from the gingival margin of the adjacent teeth. The graft thickness was measured using a periodontal probe (PCP 12 PT Hu Friedy) at 3 aspects (i.e., 1 medial and 2 lateral). If the FGG thickness was greater, the graft was thinned with the surgical blade to attain a uniform and desired thickness (i.e., 1.0–1.5 mm). The FGG was immersed in sterile saline until the placement. The donor site was sutured with a 4/0 non-resorbable PTFE monofilament (Cytoplast PTFE) to retain the clot. The FGG was positioned and sutured to the periosteum at the recipient bed with interrupted and mattress sutures with 4/0 non-resorbable monofilament material (Cytoplast PTFE) (Fig. 2).

Ten days after surgery, the sutures were removed and the sites rinsed with 0.12% chlorhexidine digluconate. After 1-, 3- and 6-months of surgery, postoperative follow-up visits were scheduled to assess the healing conditions at the donor and recipient areas. At second stage sites, the prosthetic treatment was performed 3 months after surgery.

### Clinical examination

Prior to surgery, the KT width was measured with a periodontal probe (PCP 12PT Hu Friedy) from the mucosal margin to the MGJ at the mid-vestibular aspect of the implant. The MGJ was determined by the color contrast between the KT and the alveolar mucosa. Two calibrated investigators (KM and CS) assessed the KT measurements at 1-, 3- and 6-months.

### Outcome assessments

The primary outcome was defined as 3D tissue thickness changes (mm) at the vestibular aspect of treated implant sites over the 6-month follow-up period.

Secondary outcomes included (1) changes of KT width over a 6-month, and (2) surgical time, measured to the closest minute from the start of the incision to the completion of the last suture.

### Patient-reported outcomes

Ten days after the surgery, patients were asked to fill out a form comprising 4 items: (1) evaluation of intensity of pain/discomfort after the surgery [responses scored on a visual analog scale (VAS, from 0 to 100 mm)], (2) duration of pain (in days), (3) number of analgesic tablets taken and (4) willingness to undergo the same surgery again (yes/no).

### Volumetric analysis

Intraoral digital scans of the treated area were acquired using an intraoral scanner (3 Shape Trios move, Germany GmbH) after a healing period of 1 month (S0), 3 months (S1) and 6 months (S2). To enable accurate superimposition of the scans taken at multiple points, caution was taken to capture reproducible and fixed reference points (i.e., adjacent teeth/implants).

**Fig. 3:**
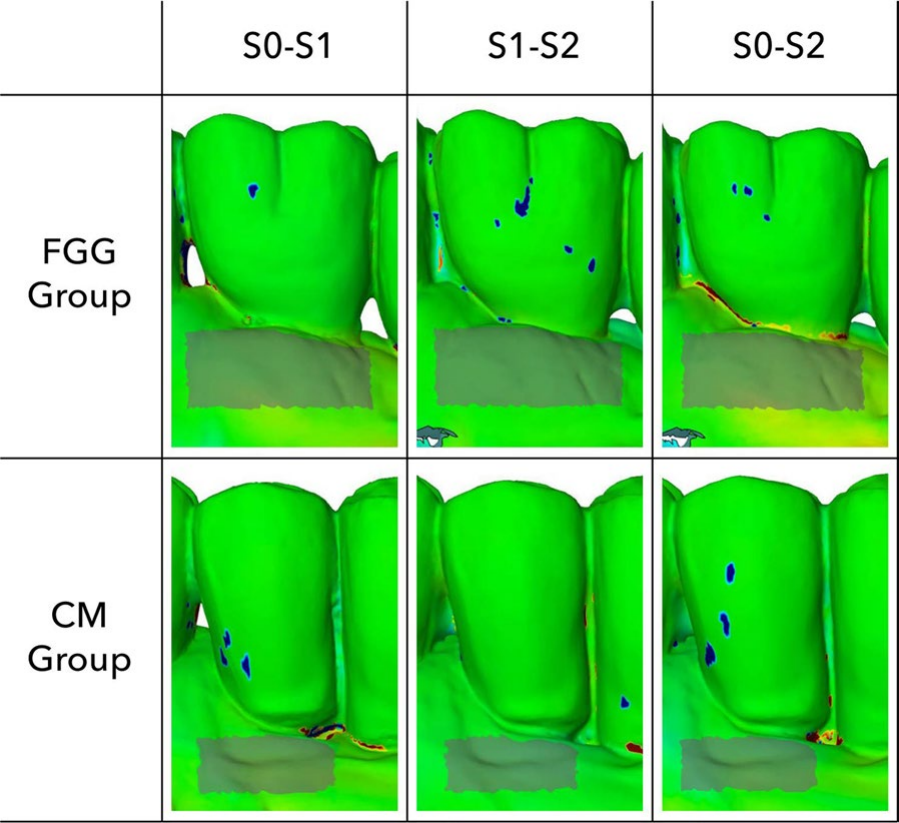
The illustration of two cases displaying the assessed volumetric measurements at the region of interest (ROI) in the CM and FGG groups at different follow-up periods

The scanned files were converted to the Standard Tessellation Language (STL) file format and exported into a software program (Meshlab, ISTI, Italy, 2016) for superimposition. The S0, S1 and S2 digital models were aligned by manually selecting at least eight reproducible points on fixed anatomical structures. The superimposed files were exported into GOM (GOM Inspect Suite 2020, Zeiss Company, Braunschweig) software for the assessment of thickness changes. To account for the grafts’ surface shrinkage, the standardized region of interest (ROI) was defined at S2. The horizontal extension of ROI corresponded to the highest mesial-distal width of the prosthetic restoration (i.e., equator area) and the vertical extension was estimated by subtracting the pre-operative KT width from the clinically measured KT width at 6-months follow-up (Fig. 3).

The thickness changes in the ROIs were recorded from S0 to S1, from S0 to S2, and from S1 to S2. For each superimposed digital model, the minimum and maximum deviation and the arithmetic mean with its standard deviation were recorded. One experienced examiner (JW) performed all measurements. Each analysis was performed in triplicate. Prior to the start of the analysis, an intra-examiner calibration was performed to determine the reproducibility of the measurements. The calibration when repeated measurements of 5 scans presented an intraclass correlation coefficient between 0.81 and 1.

### Statistical analysis

Mean values, standard deviations, medians, minimums/maximums, and 95% confidence intervals (95% CI) for primary and secondary outcomes were computed using a commercially available software program (SPSS, 27.0, Chicago, IL, USA). The analysis was carried out at implant- and patient-levels. For the patient-level analysis, in patients with more than one implant, the mean values of the multiple implants were used in the analyses.

For the patient-level analysis, unpaired t-tests were used to compare the volumetric changes (the primary outcome) between the groups at the three time points. For the secondary outcomes, between-group comparisons at the patient-level were assessed by employing the Mann–Whitney U test. To account for multiple testing, a Bonferroni correction was used leading to a significance level of 1.67%.

At the implant-level, multivariate linear regressions with mixed effects were used to assess the differences between the groups for the thickness change over time, KT width values at 1, 3 and 6 months, and changes in KT width at different time points. Nested random effects (the patient and the implant) were employed considering the treatment group (CM and FGG) and the time points as fixed effects. In order to adjust for baseline KT values as a confounder, this variable was also considered as a fixed effect in the regression.

Furthermore, a multiple linear regression analysis with mixed effects was conducted to evaluate the influence of the treatment approach (i.e., CM or FGG) and the change in KT width from S0 to S2, S1 to S2, and S0 to S2 on volumetric change at the treated implant sites.

A Shapiro–Wilk-Test with a significance level of 5% was used to assess the normality of the data. The multiple regressions included a maximum of 162 measurements (= 54 implants at 3 time points) for each dependent variable. For the normality of the residuals of the multiple regressions (sample sizes larger of at least 140 despite missing values), a significance level of 3% was chosen.

## Results

The initial study population consisted of 34 patients (17 in the CM group and 17 in the FGG-group). In the CM group, one patient (one implant) did not attend the scheduled follow-up appointments, and another patient (one implant) moved to another country; therefore, finally 15 (11 female and 4 male; mean age: 62.86 ± 14.31 years) patients with 23 implants were available for the analysis in the CM and 17 patients (6 female and 11 male; mean age: 63.40 ± 10.68 years) with 31 implants in the FGG group, respectively. Table 1 presents the demographic data and implant site characteristics.

**Table 1:** The demographic data and implant site characteristics

	CM	FGG
Patient number (n)	15	17
Female/ male (n)	11/3	6/11
Age (mean ± SD/ median) (years)	62.86 ± 14.31/61.17	63.40 ± 10.68/62.88
Implants	23	31
Region		
Upper jaw anterior anterior*/posterior	0/7	1/12
Lower jaw anterior/posterior	0/16	0/18
Time of surgery		
Second stage surgery	7 patients/11 implants	7 patients/11implants
Implants in function	8 patients/12 implants	10 patients/20 implants

Anterior*—from canine to canine

Healing was uneventful in all of the patients. Seven patients/11 implants in the CM group and 7 patients/11 implants in the FGG group underwent soft-tissue augmentation at second-stage surgery, and the remaining 8 patients/12 implants in the CM group and 10 patients/20 implants in the FGG group were treated after implant loading. Except for one implant site in the FGG group, all surgeries were performed in posterior areas.

### Dimensional assessments

**Fig. 4:**
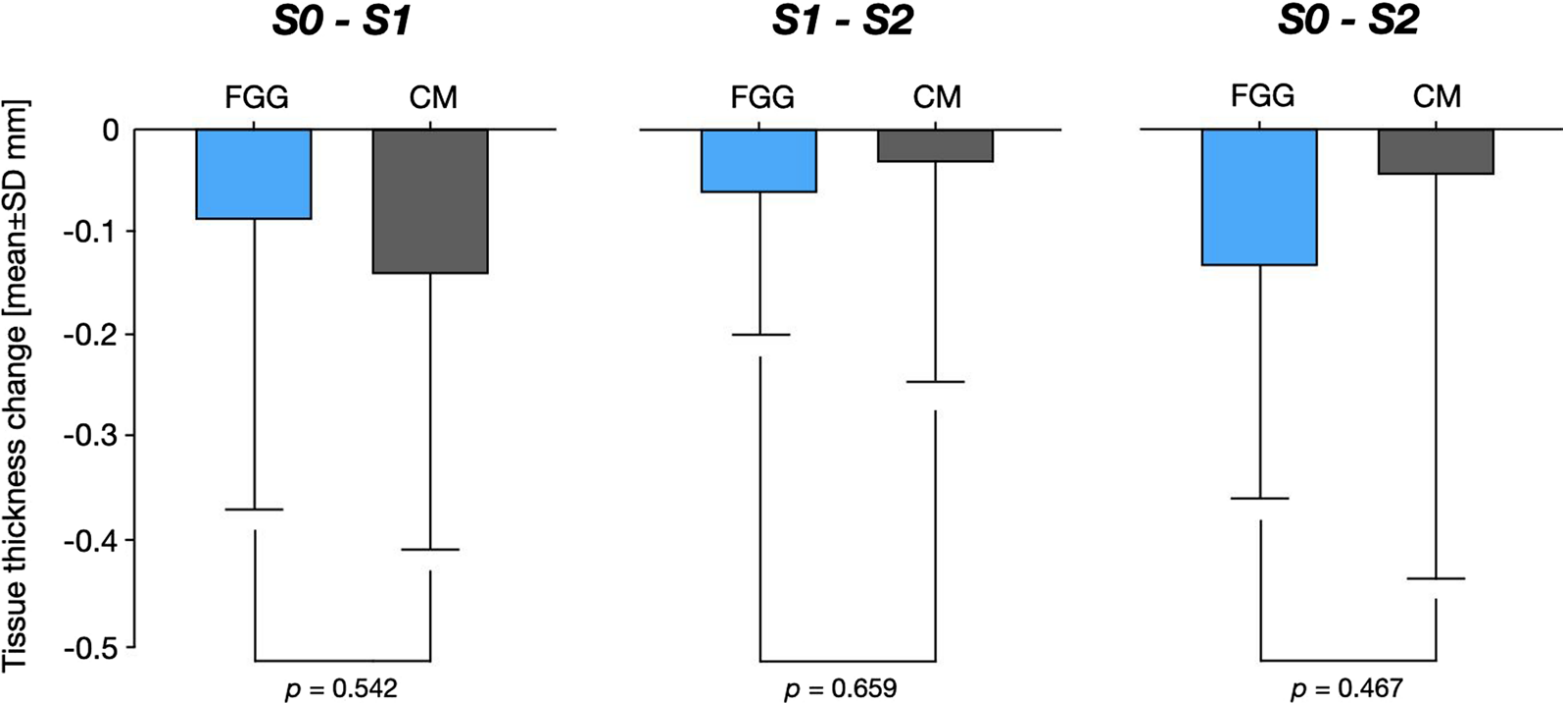
Bar diagram depicting the assessed volumetric changes in the FGG and CM groups

Table 2 and Fig. 4 represent the dimensional changes assessed in both groups over a 6-month follow-up period.

**Table 2:** Thickness changes between analyzed time intervals over a 6-month follow-up period

Patient-level	CM			FGG		
	S0–S1* (13 patients)	S0–S2** (14 patients)	S1–S2*** (14 patients)	S0–S1* (14 patients)	S0–S2** (15 patients)	S1–S2*** (16 patients)
Mean ± SD	− 0.14 ± 0.27	− 0.04 ± 0.40	− 0.03 ± 0.22	− 0.08 ± 0.29	− 0.13 ± 0.23	− 0.06 ± 0.14
95% CI	− 0.31;0.02	− 0.26; 0.18	− 0.15;0.10	− 0.24;0.09;	− 0.25;0.004	− 0.13; 0.02

Implant-level	S0–S1^§^ (21 implant)	S0–S2^33^ (22 implants)	S1–S2^333^ (22 implants)	S0–S1^3^ (26 implants)	S0–S2^33^ (28 implants)	S1–S2^333^ (29 implants)
Mean ± SD	− 0.16 ± 0.27	− 0.03 ± 0.40	− 0.05 ± 0.24	− 0.005 ± 0.30	− 0.07 ± 0.26	− 0.05 ± 0.14
95% CI	− 0.28;0.04;	− 0.21;0.14	− 0.16;0.06	− 0.13;0.12;	− 0.18;0.03	− 0.104;0.001

Between group comparison—patient level: unpaired t-test: *p = 0.542; **p = 0.467; ***p = 0.659

Implant level: multivariate linear regression with mixed effect models: ^3^p = 0.778; ^33^p = 0.016; ^333^p = 0.045

Overall, within 1 and 6 months (S0–S2), implant sites in both the CM and FGG groups showed decreases in tissue thickness. In particular, S0 to S2 estimations revealed a tissue thickness reduction of − 0.04 ± 0.40 mm (patient-level analysis) and − 0.03 ± 0.04 mm (implant-level analysis) in the CM group, and − 0.13 ± 0.23 mm (patient-level analysis) and − 0.07 ± 0.26 mm (implant-level analysis) in the FGG group. Based on the patient-level analysis, differences between the groups were not statistically significant (p = 0.467, unpaired t-test).

S0 to S1 analyses pointed to a slightly higher tissue thickness decrease in the CM group (patient-level analysis: − 0.14 ± 0.27 mm; implant-level analysis: − 0.16 ± 0.27 mm) than in the FGG group (patient-level analysis: − 0.08 ± 0.29 mm; implant-level analysis: − 0.005 ± 0.30), without reaching statistical significance between the groups (patient-level: p = 0.467; unpaired t-test; implant-level: p = 0.778, multivariate linear regression). Similarly, S1 to S2 volumetric estimations based on the patient- and implant-level analyses pointed to a mean thickness loss of − 0.03 ± 0.22 mm (patient-level) and − 0.05 ± 0.24 mm (implant-level) in the CM group, and of − 0.06 ± 0.14 mm (patient-level) and − 0.05 ± 0.14 mm (implant-level) in the FGG group, again with no significant differences noted between the groups based on the patient-level analysis (p = 0.467; unpaired t-test).

Based on the multivariate linear regression analysis with mixed effects, volumetric changes occurring between S0 and S2 were found to be significantly different between the groups, pointing to an estimated tissue volume gain of 0.076 mm in the CM group, and an estimated tissue volume loss of 0.098 m in the FGG group (p = 0.016). Likewise, when considered the volumetric tissue changes measured between S1 and S2, an estimated tissue volume gain in the CM group amounted to 0.059 mm, and the corresponding estimate in the FGG group indicated a tissue volume loss of 0.08 mm (p = 0.045).

### Clinical assessments

**Fig. 5:**
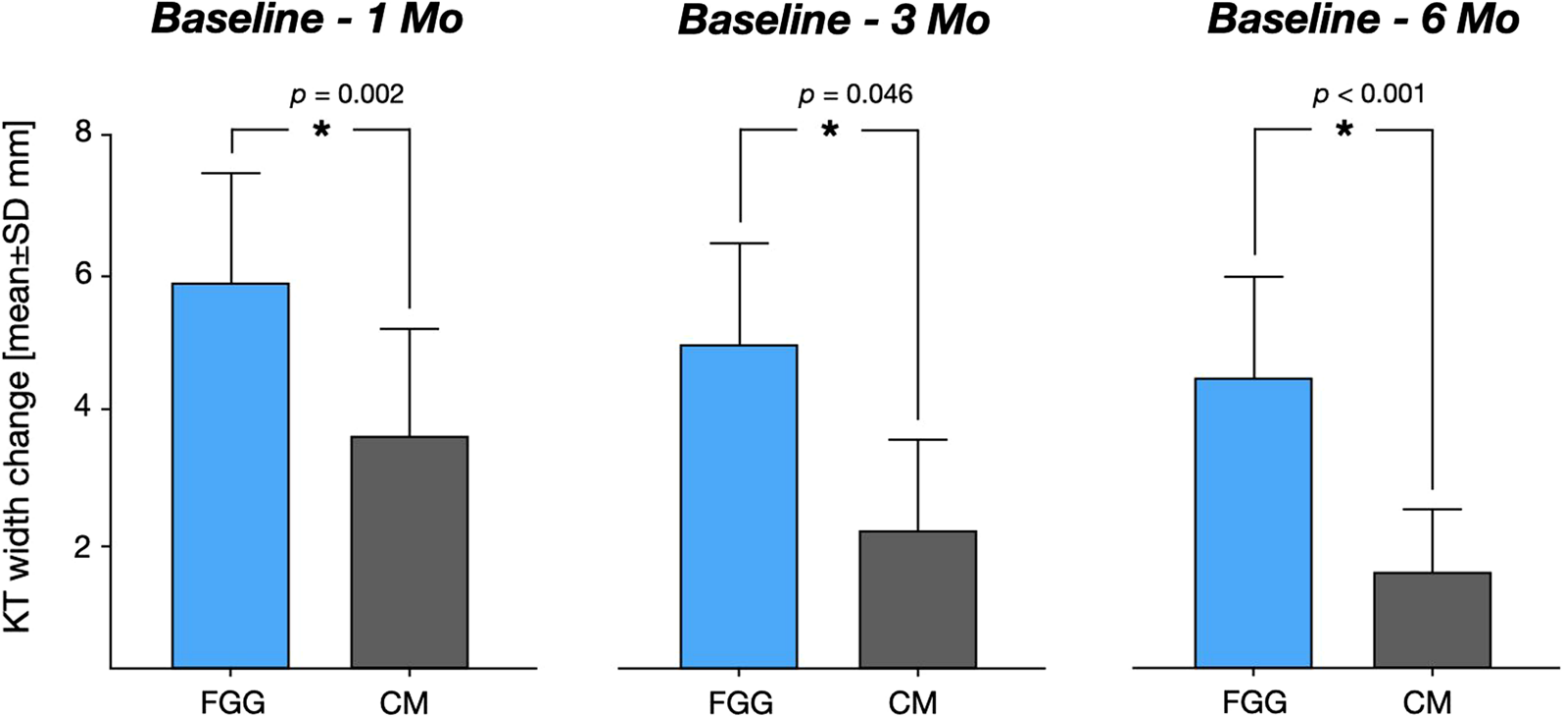
Bar diagram illustrating the KT width changes over the investigation period in the FGG and CM groups

The KT measurements at different time points and the changes in KT width over the investigation period are presented in Tables 3 and 4 and Fig. 5.

**Table 3:** The width of KT measured over a 6-month follow-up period

Patient-level	Baseline	1 month	3 months	6 months
CM	(15 patients)	(14 patients)	(14 patients)	(15 patients)
Mean ± SD	0.91 ± 0.76	4.57 ± 1.43**	3.09 ± 1.21***	2.36 ± 1.11***
95% CI	0.49;1.33	3.75; 5.39	2.39; 3.79	1.74; 2.99
FGG	(17 patients)	(15 patients)	(16 patients)	(17 patients)
Mean ± SD/median	0.34 ± 0.64*	6.29 ± 1.29**	5.27 ± 1.32***	4.86 ± 1.23***
95% CI	0.008; 0.67	5.58; 7.00	4.57; 5.98	4.23; 5.50
Implant-level				
CM	(23 implants)	(22 implants)	(22 implants)	(23 implants)
Mean ± SD	0.85 ± 0.79	4.56 ± 1.63^§^	3.07 ± 1.30^§§^	2.33 ± 1.12^§§§^
95% CI	0.51; 1.19	3.82; 5.27	2.50; 3.64	1.84; 2.81
FGG	(31 implants)	(28 implants)	(29 implants)	(31 implants)
Mean ± SD	0.29 ± 0.61	6.07 ± 1.41^§^	5.12 ± 1.30^§§^	4.71 ± 1.19^§§§^
95% CI	0.06; 0.52	5.52; 6.62	4.64; 5.63	4.27; 5.15

Between group comparison patient-level: Mann–Whitney-U test: *p = 0.028; **p = 0.003; ***p < 0.001; implant level: multivariate linear regression with mixed effect models: ^§^p < 0.001; ^§§^p = 0.120; ^§§§^p = 0.009

**Table 4:** Changes of KT width over a 6-month follow-up period

Patient-level	Change Baseline—1 month	Change 1 month—3 months	Change Baseline—3 months	Change 1-month—6 months	Change 3-months—6 months	Change Baseline—6 months
CM	(14 patients)	(13 patients)	(14 patients)	(14 patients)	(14 patients)	(15 patients)
Mean ± SD	3.66 ± 1.67*	− 1.37 ± 1.28**	2.22 ± 1.44***	− 2.11 ± 1.40****	− 0.71 ± 1.91*****	1.45 ± 1.13***
95% CI	2.7; 4.63	− 2.14; − 0.59	1.39; 3.05	− 2.92; − 1.30	− 1.77; 0.355	0.83; 2.07
FGG	(15 patients)	(14 patients)	(16 patients)	(15 patients)	(16 patients)	(17 patients)
Mean ± SD	5.90 ± 1.58*	− 1.0 ± 1.09**	4.91 ± 1.55***	− 1.38 ± 1.16****	− 0.41 ± 0.56*****	4.52 ± 1.40***
95% CI	5.02; 6.78	− 1.63; − 0.37	4.09; 5.74	− 2.01; − 0.74	− 0.71; − 0.18	3.80; 5.24
Implant-level						
CM	(22 implants)	(21 implant)	(22 implants)	(22 implants)	(22 implants)	(23 implants)
Mean ± SD	3.70 ± 1.74^§^	− 1.40 ± 1.3^$^	2.25 ± 1.53^§§^	− 2.15 ± 1.37^$$^	− 0.77 ± 0.77^§§§^	1.47 ± 1.25^$$$^
95% CI	2.93; 4.47	− 2.00; − 0.81	1.57; 2.93	− 2.77; − 1.55	− 1.11; − 0.43	0.94; 2.02
FGG	(28 implants)	(26 implants)	(29 implants)	(28 implants)	(29 implants)	(31 implants)
Mean ± SD	5.75 ± 1.62^§^	− 0.96 ± 1.04^$^	4.82 ± 1.47^§§^	− 1.35 ± 1.13^$$^	− 0.44 ± 0.63^§§§^	4.41 ± 1.31^$$$^
95% CI	5.12; 6.38	− 1.38; − 0.54	4.27; 5.38		− 0.69; − 0.21	3.94; 4.90

Between group comparison patient-level: Mann–Whitney-U test: *p = 0.002; **p = 0.0457; ***p < 0.001; ****p = 0.145; *****p = 0.136; implant level: multivariate linear regression with mixed effect models: ^§^p < 0.001; ^§§^p = 0.120; ^§§§^p = 0.009; ^$^p = 0.483; ^$$^p = 0.260; ^$$$^p = 0.810

Based on the patient-level analysis, mean baseline KT widths amounted to 0.91 ± 0.76 mm and 0.34 ± 0.64 mm in the CM and FGG groups, respectively. The corresponding values at the implant-level analysis were 0.85 ± 0.79 mm (CM group) and 0.29 ± 0.61 mm (FGG group; Table 3). According to the patient-level analysis, the mean KT width gain compared to the baseline in the CM group amounted to 3.66 ± 1.67 mm, 2.22 ± 1.44 mm and 1.45 ± 1.13 mm after 1, 3 and 6 months, respectively. The corresponding values in the FGG group were 5.90 ± 1.58 mm (1 month), 4.91 ± 1.55 mm (3 months) and 4.52 ± 1.40 mm (6 months). At all three points, a significantly higher KT width gain was measured in the FGG group than in the CM group (1 month: p = 0.002, 3 months: p = 0.0457, 6 months: p < 0.001; Mann–Whitney U-test).

At the implant-level, the mean KT width gain compared to the baseline in the CM group was 3.70 ± 1.74 mm after 1 months, 2.25 ± 1.53 mm after 3 months and 1.47 ± 1.35 mm after 6 months. In the FGG groups, the respective KT width gain was 5.75 ± 1.62 mm between baseline and 1 month, 4.82 ± 1.47 mm between baseline and 3 months and 4.41 ± 1.31 mm between baseline and 6 months. After 1 and 6 months, the between-group comparison revealed significant differences between the FGG and GM groups in favor of the FGG group (1 month: p = 0.001, 6 months: p = 0.009; multivariate linear regression with mixed models) whereas after 3 months, the differences between the groups did not reach a statistically significant level (p = 0.120).

Based on multiple linear regression analyses at implant level, an association was found between KT width changes and volumetric changes occurring between S0 and S1 in the CM group, suggesting that with each increase in KT by 1 mm there was a decrease in the tissue volume by − 0.112 mm (p = 0.008). A significant difference between the groups was observed with respect to KT width changes and volumetric changes occurring within S0 and S1, pointing to an increase in tissue volume by 0.128 mm in the FGG group with each 1 mm increase in KT when compared to the CM group (p = 0.012).

### Patients reported outcomes and surgery time

Patient reported outcomes are presented in Table 5.

**Table 5:** Patient reported outcomes

	CM	FGG	p-value
Intensity of pain (VAS 0–100)			0.370
Mean	29.40	39.59	
SD	25.33	26.33	
Duration of pain (days)			0.30
Mean	2.20	4.12	
SD	2.34	3.66	
Number of analgetic tablets			0.001*
Mean	1.20	5.65	
SD	1.08	6.39	
Willingness to undergo the same surgery again			
Yes/no	14/1	13/4	
Surgical time (min)			0.001*
Mean	23.33	39.25	
SD	7.04	10.64	

*Mann–Whitney-U test indicated a significant difference between the groups

Based on the VAS scale, the pain intensity was 29.40 ± 25.33 in the CM group and 39.59 ± 26.33 in the FGG group (p = 0.370; Mann–Whitney-U test). The pain duration was 2.20 ± 2.34 days in the CM group and 4.38 ± 3.61 days in the FGG group (p = 0.30; Mann–Whitney-U test). The number of analgesic tablets taken postoperatively was significantly lower in the CM group compared to FGG group (1.2 ± 1.08 and 5.65 ± 6.39, respectively; p = 0.001; Mann–Whitney-U test). One patient (6.7%) in the CM group and 4 (23.5%) in the FGG group indicated unwillingness to undergo the same surgical procedure again.

## Discussion

This randomized controlled clinical study aimed at assessing the 3D changes in tissue thickness at implant sites undergoing soft-tissue grafting procedures to increase KT width using either CM or FGG. Dimensional tissue thickness alterations occurring after 3 and 6 months were compared to the tissue thicknesses assessed 1 month after healing.

Based on the present data synthesis, within 1 to 6 months, implant sites in both the FGG and CM groups have been found to undergo comparable reductions in tissue thickness. A slightly higher tissue thickness decrease between 1 and 3 months was registered in the CM group, whereas a comparable tissue thickness shrinkage occurred in both groups between 3 and 6 months. A similar tendency of tissue thickness reduction after FGG augmentation at implant sites was depicted in one previous clinical investigation, where 3D thickness reductions of 38% and 33% were found at treated implant sites after 1 and 3 months, respectively, resulting in an overall tissue thickness loss of 71.8% after 3 months [6]. Nonetheless, it should be noted that the latter study used an immediate post-operative scan as a baseline, whereas in the present analysis a 1-month intraoral scan of the surgical area was utilized as a baseline, precluding a direct comparison with the findings of the former study. The results of the present volumetric analysis, on the other hand, contradict the outcomes obtained in one previous randomized clinical study, which used a probe with a rubber stop to assess tissue thickness alteration at implant sites augmented either with CM or FGG. In particular, within 1 to 6 months, both groups revealed reductions in tissue thickness, with significantly higher thickness losses registered in the CM group than in the FGG group (− 0.53 mm versus − 0.36 mm, respectively; p < 0.001) [13]. Nonetheless, it needs to be highlighted that the aforementioned study assessed one-dimensional measurements, which, again, prevent a direct comparison with the present findings. In this context, it’s worth to notice the outcomes of one recent systematic review and meta-analysis that investigated tissue thickness changes following the soft-tissue grafting aimed at increasing soft tissue thickness by using either a connective tissue graft (CTG) or substitute materials [14]. Although in terms of volumetric changes, meta-analysis estimation could not identify significant differences between the two groups (i.e., CTG versus xenogeneic collagen matrix; standardized mean difference = 0.61 mm; p = 0.11), the two-dimensional tissue thickness measurements favored the implant sites treated with autogenous grafts (i.e., CTG; weighted mean difference = 0.51 mm; p < 0.001) [14].

Upon further analysis of the current data, after 6 months, a significantly greater KT width gain was measured at implant sites treated with FGG than those treated with CM. Specifically, the mean KT gain after 6 months in the CM group was 1.47 mm, and it was 4.41 mm in the FGG group (implant-level data), pointing to a mean difference of almost 3 mm in favor of the FGG group. From the clinical perspective, the results yielded with the use of CM could be considered insufficient to achieve the goal of ending with at least 2 mm of KM around dental implants, as has been suggested in the literature [2, 15–17]. On the other hand, the previous comparative clinical studies reported on the mean KT width after 6 months at implant sites treated with FGG of 4.37 mm to 7.41 mm, whereas compared to the present findings higher values were reported for the CM group (i.e., 4.40 mm to 5.38 mm), [13, 18, 19]. Likewise, the obtained KT gain in the CM group in the current study is lower compared to the previous meta-analyses that indicated a mean KT width gain (i.e., weighted mean effect) of 2.96 mm to 3.5 mm when using substitute materials of xenogenic origin [9, 20]. Furthermore, one former systematic review with a scope to summarize the efficacy of surgical techniques for enlargements of KT at implant sites during the second-stage surgery concluded that apically positioned flap along with either FGG or xenogeneic graft materials seem to provide acceptable treatment outcomes [21]. The aforementioned discrepancies might to some extent be attributed to the difficulties in clinically assessing the boundaries of the grafted area in the CM group, as xenografts present similar color blending, contour, and texture as the adjacent area when compared to the FGG group [9]. Nonetheless, as outlined in one recent network meta-analysis, when the apically positioned flap was considered as a reference treatment, the use of FGG was the only surgical approach among the other materials (i.e., CGT, collagen matrix, acellular dermal matrix) yielding a significantly greater KT width at the implant sites (1.14 mm; p = 0.02) [20].

When further analyzing this data, it was also noted that a gradual KT width reduction occurred in both groups after 3 and 6 months, with a tendency toward greater decreases in KT width noted in the CM group. Moreover, a higher KT reduction was observed within 1 to 3 months compared to the decrease measured between 3 and 6 months. The latter findings align with those of previous clinical analyses, suggesting a higher FGG shrinkage during the early healing phase (i.e., within the first 1 to 3 months; [22, 23], and a tendency towards greater KT width shrinkage in the CM group compared to the FGG group [8]).

With respect to patient-reported outcomes, the pain intensity assessed by the visual analogue scale, as well as pain duration, tended to be lower in the CM group than in the FGG group. Similar findings were reported by previous comparative studies that associated considerably lower pain scores for the use of soft tissue substitutes than for the use of autogenous tissues [13, 19, 24]. In addition, patients in the CM group indicated a significantly lower number of analgesic medications taken postoperatively than did the patients in the FGG group. Reduced analgesic consumption was likewise indicated in one recent RCT, where FGG or CM were used for the KT establishment simultaneously with surgical peri-implantitis treatment (FGG group: 4 tablets, CM group: 2 tablets) [24]. Interestingly, in the present study, 4 patients in the FGG group and 1 in the CM group indicated an unwillingness to undergo the same surgical procedure.

The overall surgical time noted in the present study was significantly reduced in the CM group when compared with the FGG group. This result aligns with the findings of one recent systematic review and meta-analysis, which reported a surgery time for the use of soft-tissue substitutes ranging from 20 to 87 min, and from 46 to 87 min for the use of autogenous tissue, thus favoring the use of soft-tissue substitutes (n = 2 studies; WMD =  − 18.5 min.; p < 0.01) [9].

It must be disclosed that one of the major methodological limitations of the present analysis is the inclusion of patients featuring reduced KT at loaded and unloaded implants. Although the estimation of one previous meta-analysis revealed no significant correlation with regard to the timing of soft-tissue augmentation in relation to implant placement (i.e., whether augmentation took place at the second stage surgery or after implant loading), the latter aspect might have affected the accuracy of the ROI selection at the respective implant sites at different investigation time points [20]. Furthermore, the use of the 1-month scan as a baseline did not allow the assessment of the early volumetric changes at the treated implant sites (i.e., those occurring within the first 4 weeks), and subsequently the overall tissue thickness changes as compared to the preoperative situation. In fact, in the postoperative scans, the software also included the sutures in the estimations of surface thickness in the selected ROI, thereby leading to misleading measurements preventing any comparison with the scan obtained during the follow-up periods. It needs to be further stressed that in the present analysis, we did not investigate the shrinkage of the surface during the investigation period due to the similarities of the grafted area in terms of tissue structure and color in the CM group compared to the surrounding tissues, which subsequently led to difficulties in demarcating the grafted site in the respective group. On the other hand, one previous prospective study indeed depicted a significant association between the postoperative surface area of FGG and the shrinkage rate of the graft occurring within 90 days after surgery (p = 0.012) [6].

Within the limitations of the present study, it was concluded that CM and FGG were associated with comparable three-dimensional thickness changes between 1 and 6 months. While a wider KT band could be established with FGG, the use of CM significantly reduced surgical time and patients´ intake of analgesics.

## Data Availability

Not applicable.

## References

[1] Sanz M (2022). Importance of keratinized mucosa around dental implants: consensus report of group 1 of the DGI/SEPA/Osteology Workshop. Clin Oral Implants Res.

[2] Ramanauskaite A, Schwarz F, Sader R (2022). Influence of width of keratinized tissue on the prevalence of peri-implant diseases: a systematic review and meta-analysis. Clin Oral Implants Res.

[3] Thoma DS (2018). Effects of soft tissue augmentation procedures on peri-implant health or disease: a systematic review and meta-analysis. Clin Oral Implants Res.

[4] Giannobile WV (2018). Evidence-based knowledge on the aesthetics and maintenance of peri-implant soft tissues: Osteology Foundation Consensus Report Part 1-effects of soft tissue augmentation procedures on the maintenance of peri-implant soft tissue health. Clin Oral Implants Res.

[5] Griffin TJ (2006). Postoperative complications following gingival augmentation procedures. J Periodontol.

[6] Parvini P (2021). Prospective study assessing three-dimensional changes of mucosal healing following soft tissue augmentation using free gingival grafts. J Periodontol.

[7] Sanz M (2009). Clinical evaluation of a new collagen matrix (mucograft prototype) to enhance the width of keratinized tissue in patients with fixed prosthetic restorations: a randomized prospective clinical trial. J Clin Periodontol.

[8] Schmitt CM (2016). Long-term outcomes after vestibuloplasty with a porcine collagen matrix (Mucograft(®)) versus the free gingival graft: a comparative prospective clinical trial. Clin Oral Implants Res.

[9] Montero E (2022). Efficacy of soft tissue substitutes, in comparison with autogenous grafts, in surgical procedures aiming to increase the peri-implant keratinized mucosa: a systematic review. Clin Oral Implants Res.

[10] Bienz SP (2022). The influence of thin as compared to thick peri-implant soft tissues on aesthetic outcomes: a systematic review and meta-analysis. Clin Oral Implants Res.

[11] Gharpure AS (2021). Role of thin gingival phenotype and inadequate keratinized mucosa width (< 2 mm) as risk indicators for peri-implantitis and peri-implant mucositis. J Periodontol.

[12] Suárez-López Del Amo F (2016). Influence of soft tissue thickness on peri-implant marginal bone loss: a systematic review and meta-analysis. J Periodontol.

[13] Tarasenko S (2020). Comparative analysis of methods to increase the amount of keratinized mucosa before stage-two surgery: a randomized controlled study. Quintessence Int.

[14] Valles C (2022). Efficacy of soft tissue augmentation procedures on tissue thickening around dental implants: a systematic review and meta-analysis. Clin Oral Implants Res.

[15] Perussolo J (2018). Influence of the keratinized mucosa on the stability of peri-implant tissues and brushing discomfort: a 4-year follow-up study. Clin Oral Implants Res.

[16] Roccuzzo M, Grasso G, Dalmasso P (2016). Keratinized mucosa around implants in partially edentulous posterior mandible: 10-year results of a prospective comparative study. Clin Oral Implants Res.

[17] Monje A, Blasi G (2019). Significance of keratinized mucosa/gingiva on peri-implant and adjacent periodontal conditions in erratic maintenance compliers. J Periodontol.

[18] Lim HC, An SC, Lee DW (2018). A retrospective comparison of three modalities for vestibuloplasty in the posterior mandible: apically positioned flap only vs. free gingival graft vs. collagen matrix. Clin Oral Investig.

[19] Vellis J, Kutkut A, Al-Sabbagh M (2019). Comparison of xenogeneic collagen matrix vs. free gingival grafts to increase the zone of keratinized mucosa around functioning implants. Implant Dent.

[20] Tavelli L (2021). Peri-implant soft tissue phenotype modification and its impact on peri-implant health: a systematic review and network meta-analysis. J Periodontol.

[21] Bassetti RG (2016). Soft tissue augmentation procedures at second-stage surgery: a systematic review. Clin Oral Investig.

[22] Namadmalian Esfahani N (2022). Dimensional changes of keratinized mucosa after accordion versus conventional free gingival graft around dental implants: a randomized two-arm parallel clinical trial. Clin Oral Implants Res.

[23] Monje A (2022). Dimensional changes in free epithelialized gingival/mucosal grafts at tooth and implant sites: a prospective cohort study. J Periodontol.

[24] Solonko M (2022). Efficacy of keratinized mucosal augmentation with a collagen matrix concomitant to the surgical treatment of peri-implantitis: a dual-center randomized clinical trial. Clin Oral Implants Res.

