# Correction: Three-dimensional changes of a porcine collagen matrix and free gingival grafts for soft tissue augmentation to increase the width of keratinized tissue around dental implants: a randomized controlled clinical study

**DOI:** 10.1186/s40729-023-00484-0

**Published:** 2023-06-28

**Authors:** Ausra Ramanauskaite, Karina Obreja, Katharina Melissa Müller, Carla Schliephake, Johanna Wieland, Amira Begic, Iulia Dahmer, Puria Parvini, Frank Schwarz

**Affiliations:** Department of Oral Surgery and Implantology, Johann Wolfgang Goethe-University, Carolinum, 60596 Frankfurt, Germany

**Correction: International Journal of Implant Dentistry (2023) 9:13** **https://doi.org/10.1186/s40729-023-00482-2**

Following publication of the original article [1], the authors identified that the given names and family names of all authors were swapped.

The author group has been updated above and the original article has been corrected.
